# Guide: a desktop application for analysing gene expression data

**DOI:** 10.1186/1471-2164-14-688

**Published:** 2013-10-07

**Authors:** Jarny Choi

**Affiliations:** 1 The Walter and Eliza Hall Institute of Medical Research, 1G Royal Parade, Parkville, 3052, Melbourne, Australia; 2 Department of Medical Biology, The University of Melbourne, Parkville, 3010, Melbourne, Australia

**Keywords:** Data analysis, R, Gene expression, RNA-seq, Microarray, Differential expression, Software

## Abstract

**Background:**

Multiplecompeting bioinformatics tools exist for next-generation sequencing data analysis. Many of these tools are available as R/Bioconductor modules, and it can be challenging for the bench biologist without any programming background to quickly analyse genomics data. Here, we present an application that is designed to be simple to use, while leveraging the power of R as the analysis engine behind the scenes.

**Results:**

Genome Informatics Data Explorer (Guide) is a desktop application designed for the bench biologist to analyse RNA-seq and microarray gene expression data. It requires a text file of summarised read counts or expression values as input data, and performs differential expression analyses at both the gene and pathway level. It uses well-established R/Bioconductor packages such as limma for its analyses, without requiring the user to have specific knowledge of the underlying R functions. Results are presented in figures or interactive tables which integrate useful data from multiple sources such as gene annotation and orthologue data. Advanced options include the ability to edit R commands to customise the analysis pipeline.

**Conclusions:**

Guide is a desktop application designed to query gene expression data in a user-friendly way while automatically communicating with R. Its customisation options make it possible to use different bioinformatics tools available through R/Bioconductor for its analyses, while keeping the core usage simple. Guide is written in the cross-platform framework of Qt, and is freely available for use from http://guide.wehi.edu.au.

## Background

Next-generation sequencing technologies are having a massive impact on genomics [1], and challenging the research community with a wide range of data related issues. Bioinformaticians are meeting these challenges with increasing numbers of data analysis and management tools. Within the domain of RNA-seq data analysis alone, for example, multiple competing tools exist [2], each with its own strengths and weaknesses.

For the bench biologist who is keen to obtain answers to such basic questions as “which genes are differentially expressed in my dataset?” or “what is the expression profile for this gene of interest in my dataset?”, it can be challenging to navigate the landscape of available bioinformatics tools [3] without any programming background. One way to close this gap is through the use of ready-made tools designed specifically for biologists. In this article, we present a new tool that meets this challenge.

Guide (Genome Informatics Data Explorer) is a desktop application for analysing RNA-seq and microarray data. It focuses on gene centric analyses, including differential expression, gene set testing and gene annotations. The user is presented with simple-to-use graphical interfaces which leverages R [4,5] to perform the necessary bioinformatics analyses automatically. Since the vast majority of bioinformatics methods developed within the RNA-seq and microarray data analysis end up as R packages [5], Guide makes some commonly used packages such as limma [6] readily accessible to the user without having to understand the details of the package, or having to use R directly.

In addition, Guide provides the user with annotations on genes, orthologue lookups, and various other functions where data integration from different sources is required. This eliminates the often tedious task of gathering the appropriate pieces of information, transforming them into the correct formats and integrating them into the current analysis. Since R commands used by Guide can also be edited by the user, these features are designed to benefit advanced users and bioinformaticians as well as the bench biologists, and to promote easier collaborations.

The design philosophy behind Guide is to be data-centric, rather than tool-centric, and to enable the user to obtain biological meaning quickly and easily. This means that rather than presenting the user with a suite of tools, it focuses on a few selected tools with already chosen default options for a given question, and the interface is designed to flow from one set of results to another. For example, the user can go from looking at a list of differentially expressed genes in a dataset, to clicking on a gene to see its expression profile across the samples, to viewing its orthologous gene’s expression profile in another dataset. The simplicity of use does have a trade-off, however, as it comes at the expense of a reduced range of analysis options. Some applications worth mentioning in this context are MeV [7] and geWorkbench [8], both being desktop java applications highly suited for applying a large set of available analysis modules. Server based applications such as Galaxy [9] and GenePattern [10] also provide a large suite of tools and tend to be data-agnostic, with a focus on customisation of workflows. Guide can also serve as an alternative to LimmaGUI [11], which provides a graphical interface to the microarray analysis capabilities in limma.

One of the primary motivations for creating a desktop application, rather than a server-client application is for data privacy, which is a concern for many projects prior to publication. By choosing a cross-platform framework of Qt [12] for application code, we have endeavored to make the desktop application as accessible as possible across a wide range of operating systems.

While the current version of Guide officially supports only mouse and human genes, it is possible to support other species through advanced customisation options (see Data Input section for details). Being gene centric, Guide does not currently support transcript-level analysis or ChIP-seq data analysis for example, however many possibilities exist for expanding the capabilities of Guide in future versions due to its core design in which the GUI application sits in front of the R analysis engine.

## Implementation

**Figure 1:**
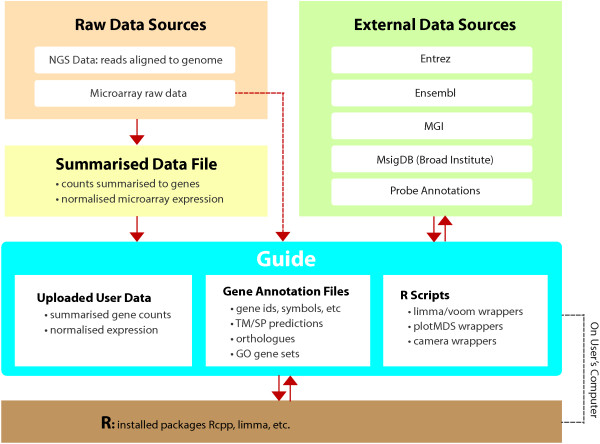
Guide design and its relationship with external resources.

Guide uses a set of relevant data files stored locally on the user’s machine, and communicates with a locally installed R instance for analyses (see Figure 1). Many of the data files are gene annotation related data, coming originally from external sources such as Entrez Gene [13], but parsed into forms suitable for use by the application. This way, the application can control when its data files should be updated, depending on the updates made in the originating data sources. Installation of R on the user’s machine is a requirement for running Guide, as is the installation of an R package called Rcpp [14], as this package enables the exchange of objects between C++ and R. Other packages such as limma and edgeR [15] used by Guide can be installed by Guide automatically as needed, provided that an internet connection exists at this point.

## Results and discussion

The example dataset included with Guide will be used to illustrate a typical workflow in this section, highlighting key features of Guide.

## Data input

The starting point for the RNA-seq data analysis is a text file of summarised read counts, where the row ids are gene ids (Entrez or Ensembl [16]) and column ids are individual sample ids (see Table 1). This means that the raw data needs to be mapped to a reference genome and the reads summarised to genes outside Guide prior to data input (see Figure 1). For microarray data, the input may be a text file of normalised expression data, with probe ids as row ids. Guide will map the probe ids to gene ids using one of its data files designed for this purpose. It currently provides this mapping for the Illumina Mouse WG-6 v2.0 array and Affymetrix MG-430 PM Array only, however the user is able to modify the mapping file if needed, by appending to the text file which contains the probe id to gene id mapping. Further explanations and help on this option is found under the Tools menu, and on the Guide website [17]. Currently Guide can also perform background correction and quantile normalisation [18] automatically for Illumina Mouse WG-6 v2.0 array, thus making it more convenient for the user by requiring only the raw data as input. These types of support for microarrays may be increased in future versions based on user demand.

**Table 1:** Example data format for input into guide

**GeneId**	**NA18486**	**NA18498**	**NA18499**
84190	6	32	14
152118	0	0	1
84321	408	475	220

Guide accepts tab-delimited text files as input data, where gene ids form row ids and sample ids form column ids. Preferred gene id is Entrez gene id, and Ensembl gene ids will be converted to Entrez ids using the gene2ensembl file from Entrez [19].

Once data is uploaded, it will keep a copy of the data so that it is readily accessible upon restarting the program. Guide comes with an example dataset, which is a subset of the example dataset used in the “RNA-Seq Case Studies” chapter of limma user’s guide [20] (this data originally comes from Pickrell et al. [21]). This example dataset can be used to try out the various functionalities without having to upload data first.

The current version of Guide officially supports mouse and human genes only. However, limited support for other species is possible in the current version using a slightly advanced customisation option, specifically by creating a text file containing the information about the genes and using the existing files as templates. This is described in more detail on the Guide website.

### Differential expression analysis

Obtaining a list of differentially expressed genes for a selected dataset is a simple matter of selecting the contrasting groups of samples, as well as changing the default normalisation and filtering options if required. In the first step, the user would define a “sample group”, with possible values assigned to each sample in the dataset appropriately. The example dataset comes with a sample group already defined, called “gender”, with “male” and “female” assigned as possible values to each sample. Guide will then programmatically use these sample groups as covariates in the linear model, as constructed by limma. The same normalisations options which are available in the calcNormFactors function of edgeR are available for selection here, including “TMM”, “RLE” and “upperquartile” [15]. Filtering can be done for lowly expressed genes by clicking on the “filter genes” link on the same page, and some sensible default values have been assigned here, which can be overwritten by the user.

**Figure 2:**
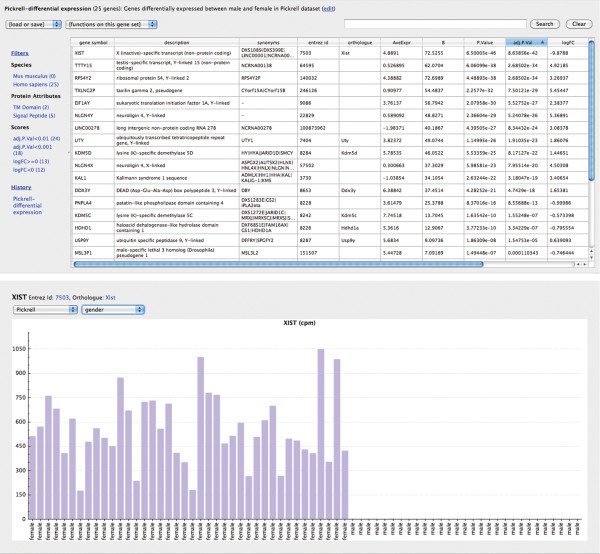
Screenshots which show results of differential expression analysis and expression profile for a selected gene.

Behind the scene, the dataset is converted to a suitable R matrix object, and vectors are created based on sample groups, which can be used to create the design matrix. R process is then called automatically to run an R script which takes these objects as input, and also acts as a wrapper to the underlying R functions. Guide currently uses the voom [22] function in the limma package for differential expression analysis, and the output of the script is a modified version of the topTable function from limma, which includes logFC and adjusted p-values. This output is then parsed by Guide into a table of genes, incorporating the available gene annotations (Figure 2).

### Gene annotation and gene set management

Gene annotation is a key feature of Guide, which has been designed to integrate data from different sources for the user’s convenience. Currently included gene annotations include synonyms and chromosome information from Entrez, transmembrane domain and signal peptide predictions from Ensembl, and mouse-human orthologues from Mouse Genome Informatics (MGI) [23]. In the current example, the resulting table from differential expression analysis shows that 24 genes were differentially expressed between males and females (this number may vary slightly depending on the normalisation and filtering options used), where the adjusted p-value (which is the p-value adjusted for multiple testing) was less than 0.05. The interesting observation from this gene set is that most genes are on the X or Y chromosomes, as indicated by the chromosome column. Clicking on the “logFC<0” filter immediately shows only the down-regulated genes, which can be seen to be mostly on the Y chromosome (provided that female vs male was chosen on the differential expression analysis page, rather than male vs female). It is therefore easy to see that Guide can create with just a few clicks, a complex query such as “show me up-regulated genes between males and females, and which of these are on the Y chromosome, and have adjusted p-value < 0.001”.

The table of genes shown can be saved to a text file, which will include all the information displayed on the screen. The same file can be used to import a gene set, thus helping collaborators share gene sets and results more easily. It is also possible to obtain a gene set by uploading a set of identifiers, hence providing a quick way to annotate an existing gene set.

Clicking on the gene symbol in this table shows the expression profile page, which can plot normalized counts per million values across the samples. This plot can group samples based on any sample groups defined, making it easier to visualise any differences. If other datasets have been uploaded into Guide, one can view the expression profile of same gene in the other dataset on this page.

Another feature available on any table of genes is the heatmap function, which can plot a heatmap for the set of genes after the user selects a dataset. Plotting the heatmap for the gene set in the current example will show a clear pattern of differential expression for these genes between the male and the female samples. We expect to refine the heatmap function in future versions to maximise its utility.

### Pathway analysis

Given any set of genes, Guide can fetch a list of enriched GO pathways, using a fisher-exact test to calculate the p-values. Running the enriched GO pathways analysis on the current example set of differentially expressed genes shows a number of GO pathways with p-value <0.05, including “histone H3-K4 demethylation”, and “regulation of chromatin silencing” under the “Process” category.

It can also perform pathway analysis against other stored sets of pathways, from the differential expression page. Currently implemented function here is the camera [24] function from limma package, which can be used to test a large number of gene sets competitively for significance within the context of specified differential expression. We plan to add the roast [25] function in future, which can test for differential expression for the genes in the set, ignoring any outside the set. Currently, the c2 and c5 gene sets from the Broad Institute [26] and their mouse orthologue sets form the stored pathways in Guide. Future version of Guide will expand on this list, as well as making it possible for the user to specify their own set of pathways to explore.

### Dataset analysis and report generation

**Figure 3:**
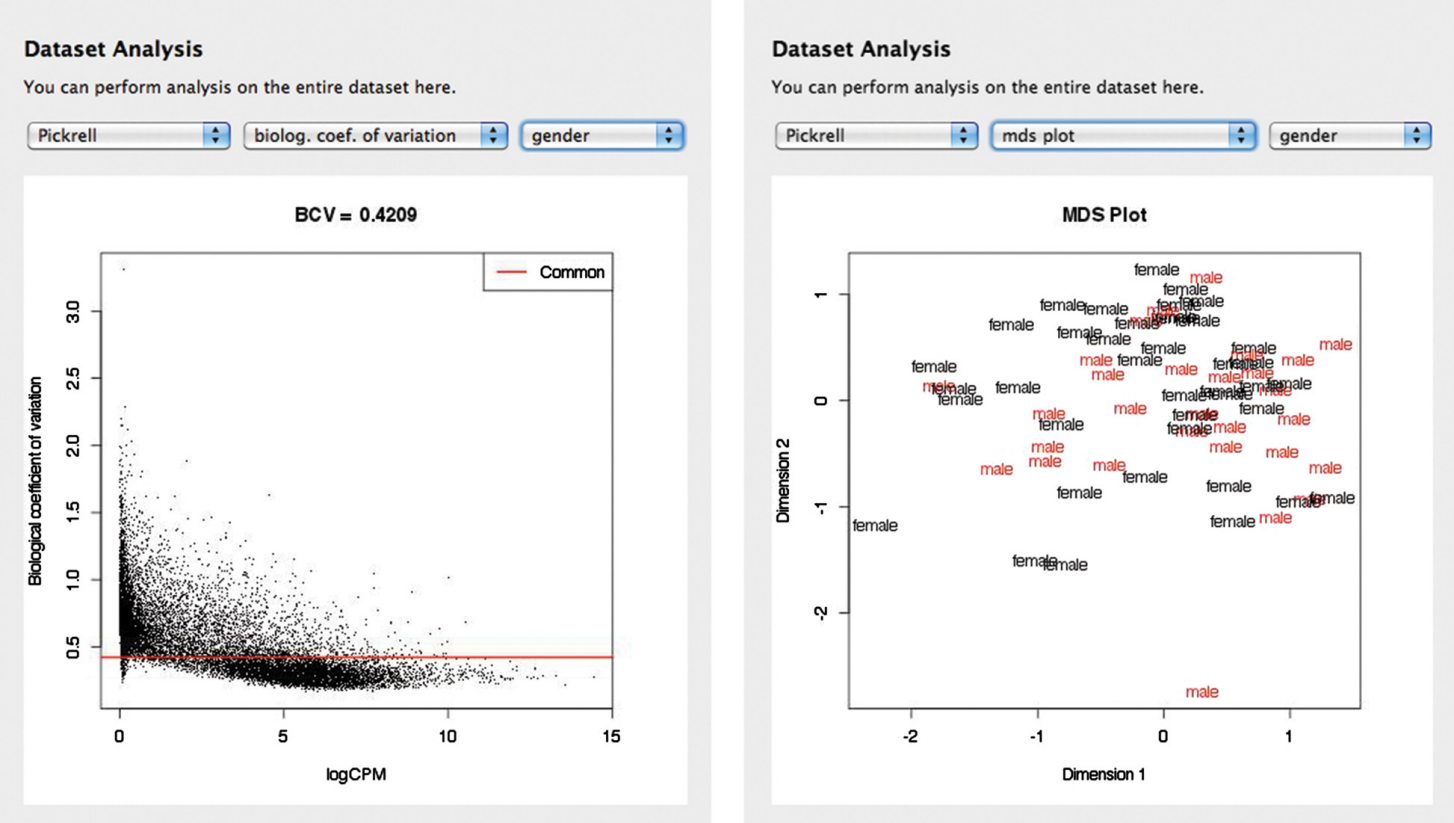
Screenshots which show available plots on the dataset, including biological coefficient of variation (plotBCV) and multi-dimentsional scaling plot (plotMDS).

Several functions work on the dataset as a whole, including a mutidimensional scaling plot (plotMDS function from limma), which performs a PCA on the dataset, and biological coefficient of variation (plotBCV function from edgeR package). Figure 3 shows these plots for the example dataset.

To support reproducibility and to make it easy to gather various analyses, Guide provides a full report generation feature on the dataset. Upon selecting various options of which analysis to include, Guide will run the appropriate R scripts to generate print quality figures, list of genes and R scripts used to generate the results, including session information which captures the versions of R and the relevant packages used for the analysis.

### Edit R scripts

Guide also provides a way for the user to view and edit the full R script used in different parts of the analysis, such as differential expression. This means that those who are familiar with R can actually change the output if desired, or save relevant objects to local files for easy transfer of data to R or other applications.

Bioinformaticians will also find Guide useful in a number of ways. One benefit is Guide’s provision of gene annotations and data integration, which alleviates the often tedious task of gathering such data from different sources manually. Another is to help collaborations with bench biologists, who are now able to explore and interact with their own data directly.

The R scripts used by Guide are not hard-coded, but accessible from the file system. This opens up the possibility of customising the scripts for particular projects, and the sharing of customised scripts by collaborators or by other bioinformatics researchers. For example, the default R script used for differential expression analysis of RNA-seq data is called “topTable.r”, and can be found amongst the data files that Guide uses (see the website for more details). If the user wishes to change the underlying function used for differential expression analysis to edgeR instead of the the default function of voom, it is only a matter of editing this file, ensuring that the function returns the correct object. Then this change will be permanent and apply to all subsequent differential expression analysis. This gives flexibility in the way that a group of collaborators may customise the analysis pipeline.

## Conclusions

Guide is a desktop application primarily designed for the bench biologist to perform gene-centric analysis on RNA-seq and microarray data without programming. Starting from a text file of summarised read counts or expression values as data input, it uses well-established R/Bioconductor packages to perform various analyses including differential expression at both the gene and pathway level, presenting the results in easy-to-use tables and figures.

While default tools and options make Guide simple to use out-of-the-box, it also contains options to customise the application for advanced users and non-standard data. An example of this is its editable R scripts feature, which can customise the R modules used for analyses and hence adapt to specific project needs. With so much bioinformatics research resulting in R modules, the key design of Guide - using R as its analysis engine - opens up many possibilities for future enhancements.

## Availability and requirements

Guide is freely available for download from http://guide.wehi.edu.au. Installation of R on the same computer is a pre-requisite for running Guide. It is written in Qt [12], and currently available for the Macintosh operating system, tested on OS >= 10.6. We are working on both the Linux and the Windows versions of the software and details can be found on the Guide website.

## Competing interests

The author declare that they have no competing interests.

## Authors’ contributions

JC conceived of and wrote the application and the manuscript.
